# Genotoxicity assessment of food-flavoring chemicals used in Japan

**DOI:** 10.1016/j.toxrep.2022.04.026

**Published:** 2022-04-27

**Authors:** Masamitsu Honma, Masami Yamada, Manabu Yasui, Katsuyoshi Horibata, Kei-ichi Sugiyama, Kenichi Masumura

**Affiliations:** a Division of Genetics and Mutagenesis, National Institute of Health Sciences, Japan; b Division of General Affairs, National Institute of Health Sciences, Japan; c Department of Applied Chemistry, National Defense Academy, Japan

**Keywords:** Food flavors, Genotoxicity, Ames test, Chromosomal aberration (CA) test, Micronucleus (MN) test, Transgenic mouse gene mutation (TG) assay, Food Safety Commission

## Abstract

We assessed the genotoxicity of 30 food-flavoring chemicals used in Japan that have not been investigated before. These 30 food-flavoring chemicals have representative chemical structures belonging to 18 chemical classes. The Ames and chromosomal aberration (CA) tests (*in vitro* tests) were first conducted in accordance with the “Food Additive Risk Assessment Guidelines” of the Japan Food Safety Commission. If the *in vitro* test yielded a positive result, an *in vivo* micronucleus test or a transgenic mouse gene mutation assay was performed to verify the *in vitro* test results. Of the 30 food-flavoring chemicals, 3 yielded a positive result in both Ames and CA tests. Another 11 chemicals yielded positive results in the CA test. However, none of the chemicals yielding positive *in vitro* test results yielded positive results in the *in vivo* tests. These findings indicate no genotoxicity concerns of the food-flavoring chemicals belonging to the abovementioned 18 chemical classes used in Japan unless there are other structural modifications.

## Highlights

• We assessed the genotoxicity of 30 food-flavoring chemicals belonging to 18 chemical classes used in Japan.
• The Ames and CA tests were first conducted, and followed-up by the *in vivo* MNtest or TG assay.
• Fourteen chemicals yielded positive results in the *in vitro* test that were negative in the *in vivo* tests.
• No genotoxicity concerns of the 18 chemical classes' food-flavorings used in Japan.

## Graphical Abstract

**Figure:**
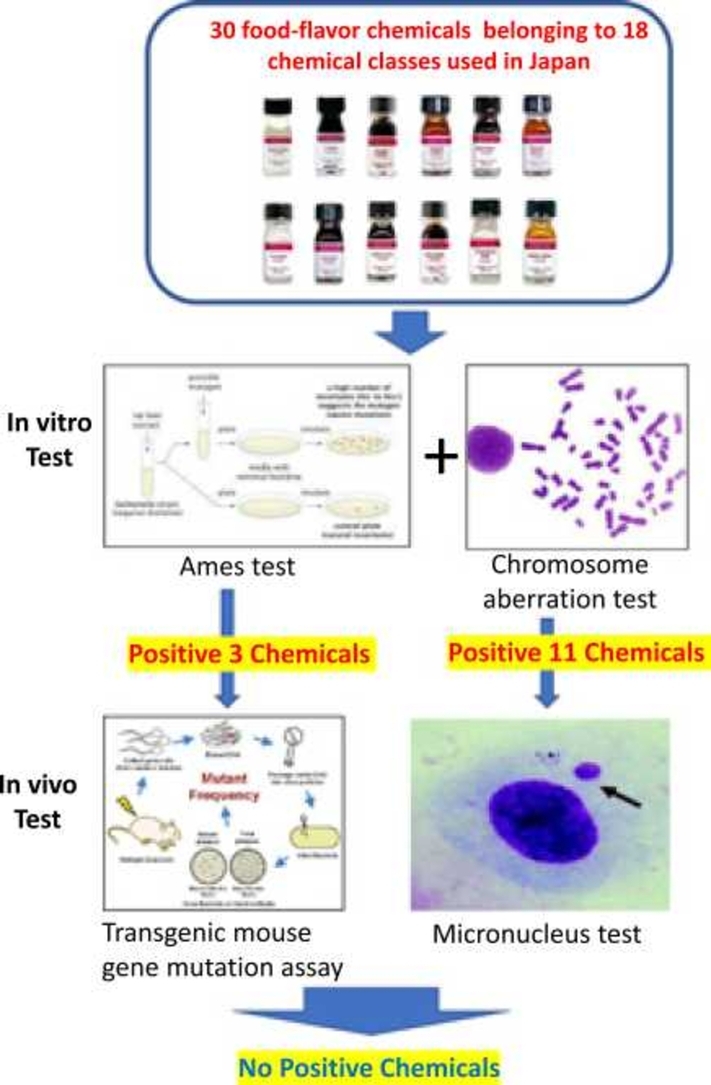

## 1 Introduction

Currently, approximately 3200 food-flavoring chemicals belonging to 18 chemical classes and designated as food additives as per the Food Sanitation Regulation can be used in Japan [19]. Although many of these chemical substances are recognized safe for use as food-flavoring agents in countries other than Japan, the safety of some chemical compounds is not adequately assured for use in Japan owing to limited data. As flavoring chemicals are present in foods at extremely low concentrations, several types of toxicities are not of concern. However, genotoxic carcinogens are thought to have no threshold, and the risk of carcinogenicity will not be zero unless the exposure level is zero [5]. For this reason, regulatory authorities in Japan, US and EU require that all food additives, including food-flavoring chemicals, be non-genotoxic to humans. Therefore, in order to ensure food safety, it is very important to properly assess the genotoxicity of food-flavoring chemicals for which the presence or absence of genotoxicity has not been confirmed. Herein, we investigated the genotoxicity of 30 food-flavoring chemicals used in Japan that have not been assessed before. These chemical substances are categorized into 18 classes based on their structural characteristics. We first conducted two *in vitro* tests, Ames test and chromosomal aberration (CA) test, according to the “Guidelines for Risk Assessment of Food Additives” established by the Food Safety Commission of Japan (FSC) [1]. The test chemicals showing positive results in the *in vitro* tests were further investigated using *in vivo* micronucleus (MN) tests or transgenic mouse gene mutation (TG) assays.

## 2 Material and methods

### 2.1 Test chemicals

Thirty flavoring chemical substances were obtained from manufacturers through the Japan Flavor & Fragrance Materials Association. The purities and suppliers of the test chemicals are shown in Table 1. These chemical substances are classified into 18 classes according to their chemical structures.

**Table 1:** Thirty food flavoring chemicals assessed for their genotoxicity in this study.

*1 SEQ; The numbers for each substance listed in the 18-class flavors list designated in Japan. *2 JECFA; The number of the item for which the safety evaluation has been completed by JECFA. *3 FEMA; FEMA GRAS number. *4 FL; The substance-specific classification numbers in EU Union List Part A. *5 Co E; The number of Flavouring substances and natural sources of flavourings issued by the Council of Europe.

### 2.2 Ames test

The Ames test was conducted by a contract research organization (CRO) in compliance with good laboratory practice (GLP) according to the Organization of Economic Co-operation and Development (OECD) guideline TG471 [17]. The test guideline requires testing using five strains (*Salmonella thyphimurium* TA100, TA98, TA1535, TA1537*,* and *Escherichia coli* WP2 *uvrA*) under both the presence and absence of rat S9 mix prepared from phenobarbital and 5,6-benzoflavone-induced rat liver. An increase of ≥ 2 times in the number of revertant colonies compared with the control in at least one Ames test strain in the presence or absence of the S9 mix indicated a positive result. Dose dependency and reproducibility were also considered in the final judgment. The maximum relative activity value (MRAV), defined as the maximum number of induced revertant colonies per mg, was calculated for all samples showing a positive result.

### 2.3 CA test

CA test using Chinese hamster lung (CHL) cells was conducted by a CRO in compliance with GLP according to the OECD guideline TG473 [15]. CHL cells were treated with a chemical for 6 h in the presence and absence of metabolic activation (short exposure) and treated with the chemical for 24 h in the absence of metabolic activation (long exposure). After the exposure, the cells were treated with a metaphase-arresting compound (colchicine) and harvested. Subsequently, chromosomal spread slides were prepared and stained. Metaphase cells were analyzed for the presence of structural and numerical aberrations (polyploidy) *via* microscopy. Negative and positive controls were included in each experiment. Positive or negative results were determined in accordance with the method described by Ishidate, in which each dose resulting in structural aberration and polyploidy at ≥ 10% is judged as positive [12]. Statistical analysis was conducted for some studies using χ2 test or Fisher’s exact test for each dose, and the difference between the test and negative control was analyzed at a one-sided significance level of 5%. In the final judgment, dose dependency was also considered in comparison with the positive control.

### 2.4 *In vivo* MN test

The *in vivo* MN test was conducted by a CRO in compliance with GLP according to the OECD guideline TG474 [16]. Animals were treated in accordance with the regulations of the Animal Care and Use Committees of the laboratories and the National Institute of Health Sciences (NIHS), Japan. Male CD1 mice (8 weeks old) were used (5 mice each for treatment, positive control, and negative control groups for main study). The dosing formulations of the test compound and negative and positive controls were orally administered daily for 2 days with a 24-h interval between the doses using stomach tubes and plastic syringes. Mice were sacrificed at 23 − 24 h after the final administration. Subsequently, femurs were removed, and bone marrow cells were prepared. According to the method described by Hayashi et al., bone marrow cell smears were prepared and observed after acridine orange fluorescent staining [2]. Polychromatic erythrocytes (PCEs) and micronucleated PCEs (MNPCEs) possessing micronuclei were counted. The frequencies of MNPCEs for each dose were statistically analyzed using Fisher’s exact test or Kastenbaum–Bowman test, and the difference between the test and negative control was analyzed at a one-sided significance level of 5%. For test substances showing significant differences, dose–response was analyzed using the Cochran–Armitage trend test.

### 2.5 TG assay

The TG assay was conducted by a CRO in compliance with GLP according to the OECD guideline TG488 [18]. Animals were treated in accordance with the regulations of the Animal Care and Use Committees of the laboratories and NIHS, Japan. Muta™ mice (CD2-LacZ80/HazfBR) aged 9 weeks were used (6 mice each for treatment, positive control, and negative control groups for main study). The dosing formulations of the test compound and negative and positive controls were administered daily for 28 days *via* oral gavage. Subsequently, 3 days after the final administration, the mice were sacrificed and their liver and glandular stomach were collected. Genomic DNA extraction was performed using the collected liver and glandular stomach (whole tissue) according to the phenol/chloroform method. Transgenes were rescued *via* an *in vitro* packaging reaction. Mutant frequency (MF) was estimated using the lacZ positive selection method [4]. MFs were statistically analyzed using Fisher’s exact test or Kastenbaum–Bowman test to compare the treated groups with the negative control group.

## 3 Results and discussion

We assessed the genotoxicity of 30 representative food-flavoring chemicals belonging to 18 classes. The assessment strategy was based on the “Guidelines for Risk Assessment of Food Additives” established by the FSC [1]. Accordingly, the Ames test and CA test were first conducted as *in vitro* tests. If the *in vitro* tests yielded a negative result, it was judged that there were no genotoxicity concerns. If the Ames test was positive, TG assay was performed to confirm the mutagenicity *in vivo*. If the CA test was positive, MN test was performed to confirm the clastogenicity *in vivo*. When both the Ames and CA tests were positive, TG assay was performed.

Table 1 summarizes the results of the Ames test, CA test, MN test, and TG assay. The Ames test data have already been reported in a previous study [14], and the raw data of the Ames test are available in the additional file of the study. The CA test, MN test, and TG assay were conducted in this study. Raw data of the CA test, MN test, and TG assay are provided as the supplement data in this study.

Among the 30 food-flavoring chemicals, 3 chemicals (2,3-pentanedione, raspberry ketone, and furfural propyleneglycol acetal) yielded positive results in the Ames and CA tests. The mutagenic activities of these chemicals were moderate considering that their MRAVs were < 1000. These three chemicals were further investigated using the TG assay, yielding negative results. Another 11 chemicals (5-hexenyl isothiocyanate, 2-methylbutyric acid, 2-hexanol, 1,3,5-undecatriene, 2-furanmethanethiol, isoeugenyl methyl ether, vanillin propyleneglycol acetal, 4-ethenyl-2-methoxyphenol, 5-methyl-2-furfural, 5-methyl-2-phenyl-2-hexenal, and 4-methylbenzaldehyde) yielded positive results in the CA test. However, their clastogenic activities were not observed in the MN test. These findings indicate no genotoxicity concerns of the 30 food-flavoring chemicals belonging to 18 classes assessed in the present study. Chemicals with these representative structures may not be genotoxic unless they have other structural modifications.

Although the FSC requires the use of Ames test and CA test for the first screening of the genotoxicity of food additives, the CA test is considered unnecessary for assessing the genotoxicity of food-flavoring chemicals. The primary reason is due to its high false-positive rate. In the present study, 14 of the 30 chemicals showed positive results in the CA test but negative results in the MN test, implying that the positive results were biologically irrelevant. The high false-positive rates of the CA test are also a problem in drug development. Positive results obtained in the CA test require follow-up in *in vivo* studies, which may lead to delays in drug development and unnecessary animal testing. The International Council for Harmonization of Pharmaceutical Regulations (ICH) genotoxicity test guideline established in 2011 (ICH-S2 (R1)) approved a new strategy that omitted *in vitro* mammalian cell tests, including the CA test, to reduce biologically irrelevant positives, but requires two *in vivo* genotoxicity tests [10]. Horibe et al. also proposed omitting the *in vitro* CA test from the standard tests performed for assessing the genotoxicity of agricultural chemicals according to their experience on risk assessment at the FSC [9].

Another reason is that Ames test is sufficient for the genotoxicity assessment of food flavoring chemicals. Mutagenicity determined using the Ames test is essential for assessing the genotoxicity of chemicals. Mutagenicity must always be considered because genotoxic carcinogens do not have a threshold. If a chemical is mutagenic, the risk of cancer cannot be zero, even at low levels [5]. On the other hand, clastogens and aneugens have thresholds and usually do not pose carcinogenic risk in humans at low levels. The ICH-M7 guideline “Assessment and control of DNA reactive (mutagenic) impurities in pharmaceuticals to limit potential carcinogenic risk” issued in 2014 requires only the Ames test for assessing the genotoxicity of impurities present at very low levels in drugs [11]. The guideline recommends the TG assay to follow-up on positive Ames test results. Like drug impurities, food flavorings are chemicals to which humans are exposed at low levels through food consumption. Therefore, the same strategy of ICH-M7 should be used to assess the genotoxicity of food-flavoring chemicals to avoid unnecessary tests. The ICH-M7 guideline also allows the use of the quantitative structure–activity relationship ((Q)SAR) approach for the initial assessment of genotoxicity instead of the Ames test. We have developed and validated some (Q)SAR tools for assessing the genotoxicity of food-flavoring chemicals, demonstrating good correlation between the (Q)SAR and Ames results [6], [7], [8], [14]. We believe that (Q)SAR will be widely accepted in the field of genotoxicity as an alternative to the Ames test in the near future.

**Fig. 1:**
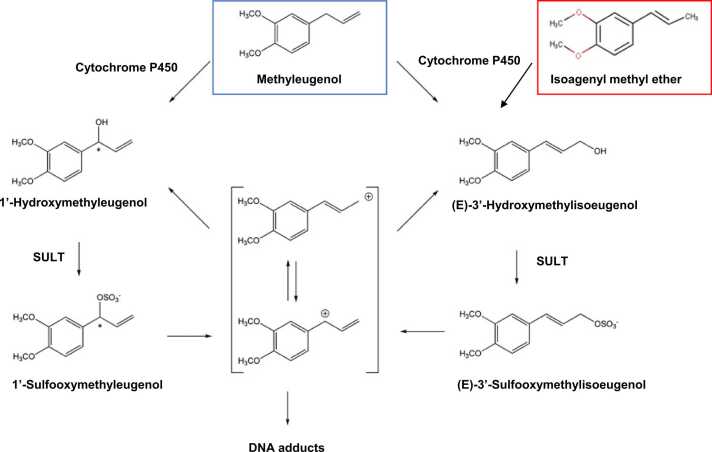
Bioactivation pathways of methyleugenol and isoeugenyl methyl ether. SULT: sulfotransferase.

However, it should be noted that the Ames test and (Q)SAR prediction may overlook the mutagenicity of some flavoring chemicals. Isoeugenyl methyl ether (phenol ether) yielded negative results in the Ames test but positive results in the CA test. However, because it yielded negative results in the MN test, we concluded that this chemical is not mutagenic or clastogenic (No. 22 in Table 1). However, a similar flavoring substance, methyleugenol, was demonstrated to be mutagenic *in vivo* in the TG assay, despite yielding negative results in the Ames test [13], [21]. Murine or human sulfotransferases (SULTs) are required to convert methyl eugenol into its mutagenic form [3]. However, rat S9 used in the Ames test lacks SULT activity. It is possible that isoeugenyl methyl ether could be metabolized in a similar manner to an active mutagen *in vivo* (Fig. 1). Hence, the TG assay is required to confirm the *in vivo* mutagenicity of isoeugenyl methyl ether. Recently, a new kinetic model considering phase II drug metabolism has been proposed, which will lead to the development of a (Q)SAR tool for predicting *in vivo* mutagenicity [20].

## 4 Conclusion

We investigated the genotoxicity of 30 representative food-flavoring chemicals belonging to 18 classes used as food additives in Japan. There are no genotoxicity concerns for all the tested chemicals. Food-flavoring chemicals belonging to these 18 chemical classes are likely to be safe for use unless they have other structural modifications.

## Funding

This work was supported by Health and Labor Sciences Research Grants (21KA1001) from the Ministry of Health, Labor and Welfare of Japan.Table 1

## CRediT authorship contribution statement

**Masamitsu Honma:** Writing – review & editing. **Masami Yamada:** Ames test data curation. **Manabu Yasui:** CA test data curation. **Katsuyoshi Horibata:** MN test data curation. **Kei-ichi Sugiyama:** Ames test data curation and supervision. **Kenichi Masumura:** TG assay data curation.

## Declaration of Competing Interest

The authors declare that they have no known competing financial interests or personal relationships that could have appeared to influence the work reported in this paper.
