# Supplementary material for: Genotoxicity assessment of food-flavoring chemicals used in Japan
Source: Toxicol Rep. 2022 Apr 27;9:1008–12. doi: 10.1016/j.toxrep.2022.04.026 (PMC9742862; doi:10.1016/j.toxrep.2022.04.026)
Supplement: Supplementary file 1 — Supplementary material [file mmc1.pdf]

Table S1-1. In vitro mammalian chromosomal aberration test of 5-hexenyl isothiocyanate (Positive)

5-hexenyl isothiocyanate (μg/mL)	Time (h)	S9 mix	Observed cells	Chromatid type: ctb	Chromatid type: cte	Chromosome type: csb	Chromosome type: cse	Others	g	RCC (%)	Structural aberrations (%)	Numerical aberrations [(b)] (%)
			Number of cells with structural chromosome aberration[(a)]									
NC	6-18	-	200	2	0	0	0	0	1	100	1.0	0.3
1.00			200	1	0	0	0	0	0	103	0.5	0.8
2.00			200	1	0	0	0	0	0	107	0.5	1.5
4.00			200	0	0	0	0	0	0	76.2	0.0	11.8*
PC: MMC			200	17	25	0	0	0	1	No data	19.5*	0.0
NC	6-18	+	200	2	0	0	0	0	0	100	1.0	1.0
3.00			200	1	1	0	0	0	1	102	1.0	0.5
6.00			200	1	0	0	0	0	0	73.1	0.5	11.3*
12.0			200	0	0	0	0	0	1	59.2	0.0	18.3*
PC: CP			200	24	56	0	0	0	4	No data	31.5*	0.3
NC	24	-	200	1	0	0	0	0	0	100	0.5	0.3
1.00			200	1	0	0	0	0	0	101	0.5	1.8*
2.00			200	0	2	0	0	0	0	69.8	1.0	17.5*
4.00			200	0	0	1	1	0	2	57.2	1.0	32.0*
PC: MMC			200	14	58	4	3	0	3	No data	33.5*	0.0

a) ctb: chromatid break, cte: chromatid exchange, csb: chromosome break, cse: chromosome exchange, other: including fragmentation, g: chromatid or chromosome gap, RCC: Relative Cell Count

b) Observed cells were 400.

NC: Negative control (DMSO), PC: Positive Control; CP: Cyclophosphamide (5.0), MMC: Mitomycin C (0.05)

*: $p < 0.05$ (significant difference by $\chi2$ test)

Table S1-2 In vitro mammalian chromosomal aberration test of Indole (Negative)

Indole (μg/mL)	Time (h)	S9 mix	Number of cells with structural chromosome aberration[(a)]							RCC (%)	Structural aberrations (%)	Numerical aberrations (%)
			Observed cells	Chromatid type		Chromosome type						
				ctb	cte	csb	cse	Others	g			
NC	6-18	-	200	0	1	0	0	0	0	100	0.5	0.0
100			200	0	0	0	0	0	0	87	0.0	0.5
200			200	1	0	0	0	0	0	75	0.5	0.5
400			200	1	0	0	0	0	1	42	0.5	0.0
PC: MMC			200	9	41	0	0	0	2	No data	23.0*	0.5
NC	6-18	+	200	0	0	0	0	0	0	100	0.0	0.0
38			200	2	8	0	0	0	0	99	4.0	0.5
75			200	1	12	0	0	0	1	62	6.0	0.5
150			200	4	13	0	0	0	0	40	7.0	0.5
300			200	2	19	0	0	0	0	25	9.5	3.0
PC: B(a)P			200	13	76	0	1	0	3	No data	39.0*	0.0
NC	24	-	200	0	0	0	0	0	0	100	0.0	0.5
25			200	0	1	0	0	0	0	91	0.5	0.5
50			200	0	0	0	0	0	0	74	0.0	0.5
100			200	0	2	0	0	0	0	57	1.0	0.0
200			200	0	2	0	0	0	0	42	1.0	1.0
PC: MMC			200	16	50	0	0	0	0	No data	28.5*	0.5

a) ctb: chromatid break, cte: chromatid exchange, csb: chromosome break, cse: chromosome exchange, other: including fragmentation, g: chromatid or chromosome gap, RCC: Relative Cell Count

NC: Negative control (DMSO), PC: Positive Control; B(a)P: Benzo[a]pyrene(20.0), MMC: Mitomycin C (0.1)

*: The frequency was more than 10%.

Table S1-3 In vitro mammalian chromosomal aberration test of Skatole（Negative）

Skatole (μg/mL)	Time (h)	S9 mix	Number of cells with structural chromosome aberration[(a)] Observed cells	Chromatid type ctb	Chromatid type cte	Chromosome type csb	Chromosome type cse	Others	g	RICC (%)	Structural aberrations (%)	Numerical aberrations (%)
NC	6-18	-	300	0	0	0	0	0	2	100	0.0	0.3
1.3			300	0	1	0	0	0	0	86	0.3	0.7
2.5			300	0	0	0	0	0	4	77	0.0	0.3
5.0			300	0	0	0	0	0	1	77	0.0	1.0
10			300	0	0	0	0	0	0	67	0.0	0.0
20			300	1	0	0	0	0	2	63	0.3	0.0
PC: MMC			300	16	107	3	2	0	3	95	39.3*	0.0
NC	6-18	+	300	0	1	0	0	0	0	100	0.3	0.3
1.3			300	0	2	0	0	0	1	76	0.7	0.0
2.5			300	0	2	0	0	0	0	76	0.7	0.0
5.0			300	0	1	0	1	0	0	78	0.7	0.3
10			300	0	1	0	0	0	0	60	0.3	0.0
20			300	0	1	0	0	0	0	55	0.3	0.0
PC: B(a)P			300	4	89	0	3	0	3	24	30.7*	0.3
NC	24	-	300	0	0	0	0	0	0	100	0.0	0.7
1.3			300	1	1	0	0	0	2	73	0.7	0.0
2.5			300	0	0	0	0	0	0	73	0.0	0.0
5.0			300	1	3	0	0	0	0	76	1.3	0.7
10			300	1	1	0	0	0	1	42	0.7	0.3
20			300	1	1	0	0	0	2	24	0.7	0.7
PC: MMC			300	11	117	1	2	0	4	39	41.7*	0.0

a) ctb: chromatid break, cte: chromatid exchange, csb: chromosome break, cse: chromosome exchange, other: including fragmentation, g: chromatid or chromosome gap, RICC: Relative Increase Cell Count

NC: Negative control (DMSO), PC: Positive Control; B(a)P: Benzo[a]pyrene(20.0) ,MMC: Mitomycin C (0.1)

*: $p < 0.05$ (significant difference by Fisher's exact test)

Table S1-4 In vitro mammalian chromosomal aberration test of Acetaldehyde diethyl acetal（Negative）

Acetaldehyde diethyl acetal (μg/mL)	Time (h)	S9 mix	Number of cells with structural chromosome aberration[(a)]							RCC (%)	Structural aberrations (%)	Numerical aberrations[(b)](%)
			Observed cells	Chromatid type		Chromosome type		Others	g			
				ctb	cte	csb	cse					
NC	6-18	-	200	4	0	0	0	0	0	100	2.0	0.0
300			200	4	0	0	0	0	1	108	2.0	0.5
600			200	0	0	0	0	0	0	101	0.0	0.0
1200			200	1	0	0	0	0	2	96.9	0.5	0.5
PC: MMC			200	17	25	0	0	0	1	No data	19.5*	0.0
NC	6-18	+	200	1	0	0	0	0	0	100	0.5	0.3
300			200	1	0	0	0	0	0	99.3	0.5	0.0
600			200	3	0	0	0	0	1	94.2	1.5	0.5
1200			200	1	0	0	0	0	0	99.1	0.5	0.0
PC: CP			200	24	56	0	0	0	4	No data	31.5*	0.3
NC	24	-	200	2	0	0	0	0	0	100	1.0	0.5
300			200	0	0	0	0	0	1	106	0.0	0.0
600			200	1	1	1	0	0	0	105	1.5	0.0
1200			200	0	0	0	0	0	0	104	0.0	1.0
PC: MMC			200	14	58	4	3	0	3	No data	33.5*	0.0

a) ctb: chromatid break, cte: chromatid exchange, csb: chromosome break, cse: chromosome exchange, other: including fragmentation, g: chromatid or chromosome gap, RCC: Relative Cell Count

b) Observed cells were 400.

NC: Negative control (DMSO), PC: Positive Control; CP: Cyclophosphamide (5.0), MMC: Mitomycin C (0.05)

*: The frequency was more than 10%.

Table S1-5 In vitro mammalian chromosomal aberration test of Hexanal propyleneglycol acetal（Negative）

Hexanal propyleneglycol acetal	Time (h)	S9 mix	Number of cells with structural chromosome aberration[(a)]							RPD	Structural aberrations (%)	Numerical aberrations [(b)](%)
			Observed	Chromatid type		Chromosome type						
(µg/mL)			cells	ctb	cte	csb	cse	Others	g	(%)		
NC	6-18	-	300	2	0	1	0	0	1	100	1.0	1.0
400			300	4	0	1	0	0	2	91.8	1.7	1.3
450			300	4	1	0	0	0	0	84.9	1.7	0.5
500			300	1	1	0	0	0	1	57.4	0.7	1.8
550			300	3	1	0	0	0	0	56.1	1.3	1.0
PC: MMC			300	17	34	0	0	0	2	No data	15.3*	0.5
NC	6-18	+	300	2	2	0	0	0	0	100	1.3	0.5
350			300	2	0	1	0	0	1	80.6	1.0	0.3
450			300	2	4	1	0	0	1	75.3	2.0	0.8
550			300	3	1	0	0	0	0	54.4	1.3	1.5
650			300	2	4	1	0	0	1	46.0	2.3	1.0
PC: CP			300	15	49	2	0	0	1	No data	20.7*	0.8
NC	24	-	300	2	0	0	0	0	1	100	0.7	0.3
350			300	5	0	0	0	0	2	83.6	1.7	0.8
400			300	3	1	0	0	0	1	87.3	1.0	1.0
450			300	6	0	0	0	0	1	46.9	2.0	1.0
PC: MMC			300	9	55	0	0	0	0	No data	21.0*	0.3

a) ctb: chromatid break, cte: chromatid exchange, csb: chromosome break, cse: chromosome exchange, other: including fragmentation, g: chromatid or chromosome gap, RPD: Relative Population Doubling

b) Observed cells were 400.

NC: Negative control (DMSO), PC: Positive Control; CP: Cyclophosphamide (5.0), MMC: Mitomycin C (0.05)

*: $p < 0.05$ (significant difference by $\chi 2$ test)

Table S1-6 In vitro mammalian chromosomal aberration test of 2,4-dimethyl-4-phenyltetrahydrofuran（Negative）

2,4-dimethyl-4-phenyltetrahydrofuran (μg/mL)	Time (h)	S9 mix	Number of cells with structural chromosome aberration[(a)]							RPD (%)	Structural aberrations (%)	Numerical aberrations[(b)] (%)
			Observed cells	Chromatid type		Chromosome type		Others	g			
				ctb	cte	csb	cse					
NC	6-18	-	300	1	0	0	0	0	0	100	0.3	0.8
335			300	1	0	0	0	0	1	75.3	0.3	2.0
402			300	3	3	0	0	0	0	71.2	2.0	1.8
482			300	2	2	2	0	0	0	41.3	2.0	1.8
PC: MMC			300	17	34	0	0	0	2	No data	15.3*	0.5
NC	6-18	+	300	2	0	1	0	0	2	100	1.0	0.5
335			300	4	0	0	0	0	0	96.6	1.3	1.0
402			300	0	0	0	0	0	0	76.7	0.0	0.5
482			300	5	0	0	0	0	1	57.2	1.7	1.5
PC: CP			300	15	49	2	0	0	1	No data	20.7*	0.8
NC	24	-	300	0	0	0	0	0	0	100	0.0	0.3
335			300	2	0	0	0	0	1	63.1	0.7	0.5
402			300	2	0	0	0	0	0	68.5	0.7	1.0
482			300	3	1	0	0	0	0	41.2	1.3	0.3
PC: MMC			300	9	55	0	0	0	0	No data	21.0*	0.3

a) ctb: chromatid break, cte: chromatid exchange, csb: chromosome break, cse: chromosome exchange, other: including fragmentation, g: chromatid or chromosome gap, RPD: Relative Population Doubling

b) Observed cells were 400.

NC: Negative control (Water for injection), PC: Positive Control; CP: Cyclophosphamide (5.0), MMC: Mitomycin C (0.05)

*: $p < 0.05$ (significant difference by $\chi 2$ test)

Table S1-7 In vitro mammalian chromosomal aberration test of Hexyl acetate（Negative）

Hexyl acetate (μg/mL)	Time (h)	S9 mix	Observed cells	Chromatid type: ctb	Chromatid type: cte	Chromosome type: csb	Chromosome type: cse	Others	g	RCC (%)	Structural aberrations (%)	Numerical aberrations (%)
			Number of cells with structural chromosome aberration[a]									
NC	6-18	-	200	0	0	0	0	0	0	100	0.0	0.0
191			200	0	1	0	0	0	0	89	0.5	0.0
286			200	0	0	0	0	0	0	76	0.0	0.0
430			200	0	0	0	0	0	0	92	0.0	0.0
644			200	1	1	0	0	0	0	58	1.0	0.0
967			115	0	0	0	0	0	0	4	0.0	0.9
PC: MMC(1)			200	16	45	0	0	0	1	78	30.0*	0.0
NC	6-18	+	200	0	0	0	0	0	0	100	0.0	0.5
430			200	0	0	0	0	0	0	83	0.0	0.0
644			200	0	0	0	0	0	0	92	0.0	0.5
967			200	0	0	0	0	0	0	78	0.0	0.5
1450			200	1	0	0	0	0	0	89	0.5	0.0
PC: CP			200	7	85	0	0	0	0	67	45.5*	0.0
NC	24	-	200	0	2	0	0	0	0	100	1.0	0.0
191			200	0	0	0	0	0	0	95	0.0	0.0
286			200	0	0	0	0	0	0	104	0.0	0.0
430			200	0	1	0	0	0	0	93	0.5	0.0
644			200	1	0	0	0	0	0	42	0.5	1.5
967			200	1	1	0	0	0	0	10	1.0	0.5
PC: MMC(2)			200	11	50	0	0	0	0	97	28.5*	0.0

a) ctb: chromatid break, cte: chromatid exchange, csb: chromosome break, cse: chromosome exchange, other: including fragmentation, g: chromatid or chromosome gap, RCC: Relative Cell Counts

NC: Negative control (DMSO), PC: Positive Control; CP: Cyclophosphamide (14.0), MMC: Mitomycin C ((1)0.075,(2)0.05)

*: The frequency was more than 10%.

Table S1-8 In vitro mammalian chromosomal aberration test of 2-butoxyethyl acetate（Negative）

2-butoxyethyl acetate (μg/mL)	Time (h)	S9 mix	Number of cells with structural chromosome aberration[(a)] Observed cells	Chromatid type ctb	cte	Chromosome type csb	cse	Others	g	RPD (%)	Structural aberrations (%)	Numerical aberrations [(b)] (%)
NC	6-18	-	300	3	0	1	0	0	1	100	1.3	0.0
264			300	2	0	0	0	0	0	89.1	0.7	0.3
660			300	1	0	0	0	0	0	104.2	0.3	0.3
1650			300	3	0	0	0	0	1	61.4	1.0	0.8
PC: MMC			300	18	22	4	1	0	0	No data	13.7*	0.0
NC	6-18	+	300	2	2	0	0	0	1	100	1.0	0.8
264			300	0	1	0	0	0	1	109.5	0.3	0.3
660			300	1	0	2	0	0	0	102.1	1.0	0.5
1650			300	2	0	1	0	0	2	95.5	1.0	0.0
PC: CP			300	21	46	5	0	0	3	No data	20.3*	0.3
NC	24	-	300	0	0	1	0	0	2	100	0.3	0.5
413			300	0	0	0	0	0	0	79.7	0.0	0.3
825			300	2	0	0	0	0	0	91.4	0.7	0.5
1650			300	1	1	2	0	0	0	84.9	1.3	0.8
PC: MMC			300	18	57	8	0	0	3	No data	24.3*	0.0

a) ctb: chromatid break, cte: chromatid exchange, csb: chromosome break, cse: chromosome exchange, other: including fragmentation, g: chromatid or chromosome gap, RPD: Relative Population Doubling

b) Observed cells were 400.

NC: Negative control (water for injuction), PC: Positive Control; CP: Cyclophosphamide (5.0), MMC: Mitomycin C (0.05)

*: $p < 0.05$ (significant difference by χ2 test)

Table S1-9 In vitro mammalian chromosomal aberration test of 2,3-pentanedione （Positive）

2,3-pentanedione (μg/mL)	Time (h)	S9 mix	Observed cells	Chromatid type: ctb	Chromatid type: cte	Chromosome type: csb	Chromosome type: cse	Others	g	RICC (%)	Structural aberrations (%)	Numerical aberrations (%)
NC	6-18	-	300	0	0	0	0	0	1	100	0.0	0.7
6.3			300	0	2	0	0	0	0	124	0.7	0.7
12.5			300	1	0	0	0	0	2	89	0.3	0.3
25.0			300	0	0	0	0	0	0	71	0.0	0.7
50.0			300	6	6	1	0	0	2	67	4.3*	0.0
PC: MMC			300	25	93	0	0	0	5	102	35.0*	0.3
NC	6-18	+	300	0	0	0	0	0	0	100	0.0	0.0
25.0			300	1	2	0	0	0	0	97	1.0	0.3
50.0			300	0	0	0	0	0	1	77	0.0	0.7
100.0			300	0	2	0	0	0	1	69	0.7	0.3
200.0			300	0	1	0	1	0	1	57	0.7	1.0
PC: B[a]P			300	9	163	0	2	0	1	40	54.3*	0.7
NC	24	-	300	0	0	0	0	0	3	100	0.0	1.0
6.3			300	0	0	0	0	0	0	82	0.0	0.3
12.5			300	2	0	0	0	0	1	70	0.7	1.7
25.0			300	2	2	0	0	0	0	57	1.3	1.0
50.0			300	7	7	0	0	0	3	52	4.7*	0.3
PC: MMC			300	18	113	0	0	0	3	77	39.7*	0.3

Number of cells with structural chromosome aberration[a]

a) ctb: chromatid break, cte: chromatid exchange, csb: chromosome break, cse: chromosome exchange, other: including fragmentation, g: chromatid or chromosome gap, RICC: Relative Increase Cell Count

NC: Negative control (physiologic saline), PC: Positive Control; B[a]P: Benzo[a]pyrene (20.0), MMC: Mitomycin C (0.1)

*: $p < 0.05$ (significant difference by Fisher's exact test)

Table S1-10 In vitro mammalian chromosomal aberration test of Raspberry ketone（Positive）

Raspberry ketone (μg/mL)	Time (h)	S9 mix	Number of cells with structural chromosome aberration[(a)] Observed cells	Chromatid type ctb	Chromatid type cte	Chromosome type csb	Chromosome type cse	Others	g	RCC (%)	Structural aberrations (%)	Numerical aberrations (%)
NC	6-18	-	200	0	0	0	0	0	0	100	0.0	0.0
482			200	1	5	0	0	0	1	68	3.0	0.0
579			200	3	7	0	0	0	0	65	4.0	0.5
694			200	3	9	0	0	0	0	61	5.5	1.5
833			200	5	17	0	0	0	0	59	9.0	1.0
1000			200	6	12	0	0	0	0	51	8.0	0.5
PC: MMC			200	14	18	0	0	0	0	60	14.0*	0.0
NC	6-18	+	200	0	0	0	0	0	0	100	0.0	0.0
24.1			200	2	12	0	0	0	1	68	6.5	1.5
28.9			200	2	21	0	0	0	0	64	10.5*	0.5
34.7			200	13	36	0	0	0	0	62	21.0*	0.5
41.7			200	17	48	0	0	0	0	51	27.0*	0.5
50.0			200	39	76	0	0	0	0	51	47.0*	0.0
PC: CP			200	36	108	0	0	0	0	60	61.5*	0.0

a) ctb: chromatid break, cte: chromatid exchange, csb: chromosome break, cse: chromosome exchange, other: including fragmentation, g: chromatid or chromosome gap, RCC: Relative Cell Count

NC: Negative control (DMSO), PC: Positive Control; CP: Cyclophosphamide (14.0), MMC: Mitomycin C (0.075)

*: The frequency was more than 10%.

Table S1-11 In vitro mammalian chromosomal aberration test of 2-methylbutyric acid（Positive）

2-methylbutyric acid (μg/mL)	Time (h)	S9 mix	Observed cells	Chromatid type: ctb	Chromatid type: cte	Chromosome type: csb	Chromosome type: cse	Others	g	RICC (%)	Structural aberrations (%)	Numerical aberrations (%)
			Number of cells with structural chromosome aberration[(a)]									
NC	6-18	-	300	0	1	0	0	0	0	100	0.3	0.3
255			300	0	0	0	0	0	0	102	0.0	0.3
511			300	0	0	0	0	0	1	100	0.0	0.3
1021			300	1	4	0	0	0	2	96	1.7	0.3
PC: MMC			300	21	113	1	0	0	8	91	39.7*	1.0
NC	6-18	+	300	0	2	0	0	0	1	100	0.7	0.0
255			300	0	0	0	0	0	2	107	0.0	0.7
511			300	0	0	0	0	0	0	109	0.0	1.3
1021			300	0	1	0	0	0	0	100	0.3	1.3
PC: B[a]P			300	6	89	0	6	0	2	62	31.7*	0.7
NC	24	-	300	1	2	0	0	0	1	100	0.7	0.7
255			300	1	0	0	0	0	0	103	0.3	1.0
511			300	1	2	0	0	0	2	108	1.0	0.0
1021			300	15	9	0	0	0	8	80	7.7*	0.3
PC: MMC			300	13	127	2	1	0	6	50	46.0*	0.0

a) ctb: chromatid break, cte: chromatid exchange, csb: chromosome break, cse: chromosome exchange, other: including fragmentation, g: chromatid or chromosome gap, RICC: Relative Increase Cell Count

NC: Negative control (physiologic saline), PC: Positive Control; B[a]P: Benzo[a]pyrene (20.0), MMC: Mitomycin C (0.1)

*: $p < 0.05$ (significant difference by Fisher's exact test)

Table S1-12 In vitro mammalian chromosomal aberration test of (5 or 6)-decenoic acid（Negative）

(5 or 6)-decenoic acid (μg/mL)	Time (h)	S9 mix	Number of cells with structural chromosome aberration[(a)] Observed cells	Chromatid type ctb	Chromatid type cte	Chromosome type csb	Chromosome type cse	Others	g	RCC (%)	Structural aberrations (%)	Numerical aberrations (%)
NC	6-18	-	200	1	0	0	0	0	0	100	0.5	0.0
56.3			200	0	0	0	0	0	0	91	0.0	0.0
113			200	0	0	0	0	0	0	97	0.0	0.5
225			200	0	1	0	0	0	0	91	0.5	0.0
450			200	0	0	0	0	0	0	58	0.0	2.0
PC: MMC[(1)]			200	3	34	0	0	0	0	79	18.5*	0.0
NC	6-18	+	200	0	0	0	0	0	0	100	0.0	0.0
113			200	0	0	0	0	0	0	97	0.0	0.5
225			200	0	0	0	0	0	0	94	0.0	0.0
450			200	0	1	0	0	0	0	70	0.5	1.0
900			200	0	2	0	0	0	1	52	1.0	0.5
PC: CP			200	7	39	0	0	0	0	84	22.5*	0.0
NC	24	-	200	0	0	0	0	0	0	100	0.0	0.0
56.3			200	1	0	0	0	0	0	101	0.5	0.0
113			200	0	0	0	0	0	0	83	0.0	0.0
225			200	0	0	0	0	0	0	76	0.0	0.5
450			200	0	0	0	0	0	0	54	0.0	0.0
PC: MMC[(2)]			200	3	46	0	0	0	0	103	23.5*	0.0

a) ctb: chromatid break, cte: chromatid exchange, csb: chromosome break, cse: chromosome exchange, other: including fragmentation, g: chromatid or chromosome gap, RCC: Relative Cell Count

NC: Negative control (DMSO), PC: Positive Control; CP: Cyclophosphamide (14.0), MMC: Mitomycin C ((1)0.075,(2)0.05)

*: The frequency was more than 10%.

Table S1-13 In vitro mammalian chromosomal aberration test of 2-hexenol（Positive）

Trans-2-hexenol (μg/mL)	Time (h)	S9 mix	Number of cells with structural chromosome aberration[a] Observed cells	Chromatid type ctb	cte	Chromosome type csb	cse	Others	g	RCC (%)	Structural aberrations (%)	Numerical aberrations (%)
NC	6-18	-	200	0	0	0	0	0	0	100	0.0	0.0
311			200	0	0	0	0	0	0	89	0.0	3.5
467			200	0	0	0	0	0	0	82	0.0	1.5
700			200	0	0	0	0	0	0	72	0.0	2.5
1050			200	0	1	0	0	0	0	49	0.5	1.0
PC: MMC(1)			200	5	34	0	0	0	0	93	19.0*	0.5
NC	6-18	+	200	0	0	0	0	0	0	100	0.0	0.0
41.0			200	0	0	0	0	0	0	94	0.0	0.0
61.5			200	0	0	0	0	0	0	95	0.0	0.0
92.2			200	1	0	0	0	0	0	76	0.5	0.0
138			200	21	55	0	1	0	0	48	34.0*	0.0
PC: CP			200	12	95	0	0	0	0	69	51.0*	0.0
NC	24-0	-	200	0	0	0	0	0	0	100	0.0	0.5
207			200	1	0	0	0	0	0	90	0.5	3.0
311			200	0	0	0	0	0	0	71	0.0	3.0
467			200	2	3	0	0	0	0	59	2.5	0.5
700			200	30	21	0	0	0	0	47	23.5*	0.0
1050			200	86	9	0	0	0	0	32	45.5*	0.0
PC: MMC(2)			200	8	53	0	0	0	0	91	29.5*	0.0

a) ctb: chromatid break, cte: chromatid exchange, csb: chromosome break, cse: chromosome exchange, other: including fragmentation, g: chromatid or chromosome gap, RCC: Relative Cell Count

NC: Negative control (DMSO), PC: Positive Control; CP: Cyclophosphamide (14.0), MMC: Mitomycin C ((1)0.075,(2) 0.05)

*: The frequency was more than 10%.

Table S1-14 In vitro mammalian chromosomal aberration test of 2-(l-menthoxy)ethanol（Negative）

2-(l-menthoxy)ethanol (μg/mL)	Time (h)	S9 mix	Number of cells with structural chromosome aberration[(a)]							RPD (%)	Structural aberrations (%)	Numerical aberrations [(b)] (%)
			Observed cells	Chromatid type		Chromosome type		Others	g			
				ctb	cte	csb	cse					
NC	6-18	-	300	3	0	0	0	0	0	100	1.0	0.3
1250			300	1	0	0	0	0	1	80.6	0.3	0.8
1400			300	2	0	0	0	0	0	66.7	0.7	0.0
1550			300	2	0	0	0	0	2	73.5	0.7	1.0
1700			300	1	2	0	0	0	0	37.8	1.0	0.3
PC: MMC			300	19	18	3	1	0	0	No data	13.3*	0.5
NC	6-18	+	300	1	1	0	0	0	0	100	0.7	1.0
1250			300	1	0	0	0	0	0	86.7	0.3	0.3
1400			300	1	2	1	0	0	1	74.3	1.3	0.5
1550			300	3	1	1	0	0	1	73.0	1.7	0.3
1700			300	1	1	1	0	0	2	46.4	1.0	0.8
PC: CP			300	25	45	1	1	0	2	No data	22.0*	0.0
NC	24	-	300	3	1	0	0	0	0	100	1.3	0.0
1250			300	2	0	0	0	0	1	79.4	0.7	0.0
1400			300	1	1	0	0	0	1	65.5	0.7	0.3
1550			300	1	0	0	0	0	0	56.7	0.3	0.8
1700			300	0	0	0	0	0	0	55.7	0.0	0.0
PC: MMC			300	18	48	0	0	0	3	No data	21.3*	0.5

a) ctb: chromatid break, cte: chromatid exchange, csb: chromosome break, cse: chromosome exchange, other: including fragmentation, g: chromatid or chromosome gap, RPD: Relative Population Doubling

b) Observed cells were 400.

NC: Negative control (water for injection), PC: Positive Control; CP: Cyclophosphamide (5.0), MMC: Mitomycin C (0.05)

*: $p < 0.05$ (significant difference by $\chi2$ test)

Table S1-15 In vitro mammalian chromosomal aberration test of Linalool oxide (furanoid)（Negative）

Linalool oxide (furanoid) (μg/mL)	Time (h)	S9 mix	Number of cells with structural chromosome aberration[(a)]							RICC (%)	Structural aberrations (%)	Numerical aberrations (%)
			Observed cells	Chromatid type		Chromosome type		Others	g			
				ctb	cte	csb	cse					
NC	6-18	-	300	1	0	0	0	0	2	100	0.3	0.3
426			300	0	0	0	0	0	0	98	0.0	0.7
851			300	2	0	0	0	0	0	93	0.7	0.3
1703			300	2	0	0	0	0	0	82	0.7	0.3
PC: MMC			300	22	108	2	2	0	2	81	40.3*	0.3
NC	6-18	+	300	0	0	0	0	0	0	100	0.0	0.3
426			300	0	1	0	0	0	0	84	0.3	0.0
851			300	0	1	0	0	0	1	76	0.3	0.0
1703			300	0	0	0	0	0	0	82	0.0	0.7
PC: B[a]P			300	13	170	0	2	0	1	32	57.0*	0.0
NC	24	-	300	0	0	0	0	0	0	100	0.0	0.0
426			300	0	0	0	0	0	0	88	0.0	0.7
851			300	0	1	0	0	0	0	80	0.3	0.0
1703			300	2	0	0	0	0	0	86	0.7	0.0
PC: MMC			300	31	132	5	0	0	5	89	49.0*	0.0

a) ctb: chromatid break, cte: chromatid exchange, csb: chromosome break, cse: chromosome exchange, other: including fragmentation, g: chromatid or chromosome gap, RICC: Relative Increase Cell Count

NC: Negative control (physiologic saline), PC: Positive Control; B[a]P: Benzo[a]pyrene (20.0), MMC: Mitomycin C (0.1)

*: $p < 0.05$ (significant difference by Fisher's exact test)

Table S1-16 In vitro mammalian chromosomal aberration test of 2-ethylbutanal（Negative）

2-ethylbutanal (μg/mL)	Time (h)	S9 mix	Number of cells with structural chromosome aberration[(a)]							RCC (%)	Structural aberrations (%)	Numerical aberrations[(b)] (%)
			Observed cells	Chromatid type		Chromosome type		Others	g			
				ctb	cte	csb	cse					
NC	6-18	-	200	1	0	0	0	0	0	100	0.5	0.0
250			200	0	0	0	0	0	1	85.5	0.0	0.3
500			200	1	0	0	0	0	0	86.9	0.5	0.3
1000			200	2	2	0	0	0	0	87.4	2.0	0.0
PC: MMC			200	10	16	0	0	0	0	No data	12.0*	0.3
NC	6-18	+	200	2	1	0	0	0	1	100	1.5	0.3
250			200	0	1	0	0	0	0	86.7	0.5	0.5
500			200	3	0	0	0	0	1	98.9	1.5	0.5
1000			200	2	0	1	0	0	0	97.8	1.5	0.5
PC: CP			200	34	79	2	0	0	0	No data	43.0*	0.3
NC	24	-	200	1	0	0	0	0	0	100	0.5	1.3
250			200	2	0	0	0	0	0	90.6	1.0	0.0
500			200	0	0	0	0	0	0	103.5	0.0	0.5
1000			200	1	1	0	0	0	0	87.2	1.0	0.3
PC: MMC			200	17	59	0	0	0	2	No data	33.0*	0.0

a) ctb: chromatid break, cte: chromatid exchange, csb: chromosome break, cse: chromosome exchange, other: including fragmentation, g: chromatid or chromosome gap, RCC: Relative Cell Count

b) Observed cells were 400.

NC: Negative control (DMSO), PC: Positive Control; CP: Cyclophosphamide (5.0), MMC: Mitomycin C (0.05)

*: The frequency was more than 10%.

Table S1-17 In vitro mammalian chromosomal aberration test of 1,3,5-undecatriene（Positive）

1,3,5-undecatriene (μg/mL)	Time (h)	S9 mix	Number of cells with structural chromosome aberration[a] Observed cells	Chromatid type ctb	cte	Chromosome type csb	cse	Others	g	RCC (%)	Structural aberrations (%)	Numerical aberrations[b] (%)
NC	6-18	-	200	0	0	0	0	0	0	100	0.0	0.3
17.9			200	3	0	0	0	0	0	85.3	1.5	0.3
23.2			200	3	0	0	0	0	0	89.8	1.5	0.3
30.2			200	0	0	1	0	0	0	86.2	0.5	0.0
39.2			200	2	0	0	0	0	0	38.8	1.0	0.0
PC: MMC			200	14	13	1	0	0	0	No data	13.0*	0.3
NC	6-18	+	200	1	1	0	0	0	0	100	1.0	0.0
46.4			200	8	1	0	0	0	1	78.8	4.5*	0.3
60.4			200	3	6	0	0	0	0	74.1	4.0	0.3
78.5			200	13	23	1	0	0	1	51.4	14.5*	1.3
PC: CP			200	34	90	0	1	0	0	No data	49.0*	0.3
NC	24	-	200	3	0	0	0	0	0	100	1.5	0.8
32.0			200	1	0	0	0	0	0	90.2	0.5	0.3
36.0			200	2	0	0	0	0	0	90.0	1.0	0.3
40.0			200	1	0	0	0	0	0	40.5	0.5	0.0
PC: MMC			200	18	39	0	2	0	0	No data	25.0*	0.3

a) ctb: chromatid break, cte: chromatid exchange, csb: chromosome break, cse: chromosome exchange, other: including fragmentation, g: chromatid or chromosome gap, RCC: Relative Cell Count

b) Observed cells were 400.

NC: Negative control (DMSO), PC: Positive Control; CP: Cyclophosphamide (5.0), MMC: Mitomycin C (0.05)

*: $p < 0.05$ (significant difference by $\chi 2$ test)

Table S1-18 In vitro mammalian chromosomal aberration test of Dimethyl sulfide（Negative）

Dimethyl sulfide (μg/mL)	Time (h)	S9 mix	Number of cells with structural chromosome aberration[(a)] Observed cells	Chromatid type ctb	cte	Chromosome type csb	cse	Others	g	RCC (%)	Structural aberrations (%)	Numerical aberrations[(b)] (%)
NC	6-18	-	200	2	0	0	0	0	2	100	1.0	0.3
163			200	0	0	1	0	0	0	98.1	0.5	0.0
325			200	0	0	0	0	0	0	98.7	0.0	0.0
650			200	2	0	0	0	0	0	99.0	1.0	0.5
PC: MMC			200	17	26	0	0	0	0	No data	19.5*	0.0
NC	6-18	+	200	1	2	0	0	0	1	100	1.5	0.3
163			200	2	0	0	0	0	1	93.7	1.0	0.0
325			200	1	1	0	0	0	1	94.9	1.0	0.0
650			200	0	1	0	0	0	1	79.9	0.5	0.3
PC: CP			200	17	58	2	1	0	1	No data	32.0*	0.5
NC	24	-	200	0	0	0	0	0	0	100	0.0	0.0
163			200	0	0	0	0	0	0	99.5	0.0	0.0
325			200	0	0	0	0	0	1	98.5	0.0	0.3
650			200	0	0	0	0	0	0	97.3	0.0	0.0
PC: MMC			200	9	46	0	0	1	1	No data	26.5*	0.5

a) ctb: chromatid break, cte: chromatid exchange, csb: chromosome break, cse: chromosome exchange, other: including fragmentation, g: chromatid or chromosome gap, RCC: Relative Cell Count

b) Observed cells were 400.

NC: Negative control (water for injection), PC: Positive Control; CP: Cyclophosphamide (5.0), MMC: Mitomycin C (0.05)

*: The frequency was more than 10%.

Table S1-19 In vitro mammalian chromosomal aberration test of 2-furanmethanethiol（Positive）

2-furanmethanethiol (μg/mL)	Time (h)	S9 mix	Number of cells with structural chromosome aberration[a] Observed cells	Chromatid type ctb	Chromatid type cte	Chromosome type csb	Chromosome type cse	Others	g	RCC (%)	Structural aberrations (%)	Numerical aberrations (%)
NC	6-18	-	200	2	1	0	0	0	1	100	1.5	0.0
4.45			200	3	0	0	0	0	0	94.2	1.5	0.5
8.91			200	0	0	0	0	0	0	57.9	0.0	1.5
17.8			200	1	4	0	0	0	0	42.1	2.0	4.0
PC: MMC			200	97	84	0	0	0	4	No data	61.5*	0.5
NC	6-18	+	200	1	1	0	0	0	1	100	0.5	0.0
35.0			200	5	6	0	0	0	0	64.9	5.0	1.0
40.0			200	57	34	0	0	0	2	55.9	38.5*	0.5
45.0			200	112	66	0	0	0	7	35.2	63.0*	0.5
PC: CP			200	21	36	0	0	0	0	No data	26.0*	0.0

a) ctb: chromatid break, cte: chromatid exchange, csb: chromosome break, cse: chromosome exchange, other: including fragmentation, g: chromatid or chromosome gap, RCC: Relative Cell Count

NC: Negative control (DMSO), PC: Positive Control; CP: Cyclophosphamide (6.0), MMC: Mitomycin C (0.1)

*: The frequency was more than 10%.

Table S1-20 In vitro mammalian chromosomal aberration test of Gamma-terpinene（Negative）

Gamma-terpinene (μg/mL)	Time (h)	S9 mix	Number of cells with structural chromosome aberration[(a)] Observed cells	Chromatid type ctb	Chromatid type cte	Chromosome type csb	Chromosome type cse	Others	g	RCC (%)	Structural aberrations (%)	Numerical aberrations (%)
NC	6-18	-	200	0	0	0	0	0	0	100	0.0	0.0
198			200	0	0	0	0	0	0	67	0.0	0.0
296			200	0	4	0	0	0	0	58	2.0	0.5
444			200	0	0	0	0	0	0	53	0.0	0.0
667			200	1	2	0	0	0	0	49	1.5	0.0
PC: MMC[(1)]			200	12	46	0	0	0	0	100	29.0*	0.0
NC	6-18	+	200	0	0	0	0	0	0	100	0.0	0.0
350			200	0	0	0	0	0	0	90	0.0	0.0
700			200	1	0	0	0	0	0	89	0.5	0.0
1400			200	0	0	0	0	0	0	92	0.0	0.0
PC: CP			200	4	141	0	0	0	0	47	72.0*	0.0
NC	24-0	-	200	1	0	0	0	0	0	100	0.5	0.0
87.8			200	0	1	0	0	0	0	101	0.5	0.0
132			200	0	1	0	0	0	0	81	0.5	0.0
198			200	0	0	0	0	0	0	81	0.0	0.0
296			200	0	1	0	0	0	0	37	0.5	0.0
PC: MMC[(2)]			200	10	57	0	0	0	0	82	32.5*	0.0

a) ctb: chromatid break, cte: chromatid exchange, csb: chromosome break, cse: chromosome exchange, other: including fragmentation, g: chromatid or chromosome gap, RCC: Relative Cell Count

NC: Negative control (acetone), PC: Positive Control; CP: Cyclophosphamide (14.0), MMC: Mitomycin C ((1)0.075,(2) 0.05)

*: The frequency was more than 10%.

Table S1-21 In vitro mammalian chromosomal aberration test of Isoeugenyl methyl ether（Positive）

Isoeugenyl methyl ether (μg/mL)	Time (h)	S9 mix	Number of cells with structural chromosome aberration[(a)]							RCC (%)	Structural aberrations (%)	Numerical aberrations (%)
			Observed	Chromatid type		Chromosome type						
			cells	ctb	cte	csb	cse	Others	g			
NC	6-18	-	200	0	0	0	0	0	0	100	0.0	0.0
59.3			200	0	0	0	0	0	0	87	0.0	0.0
88.9			200	1	0	0	0	0	0	84	0.5	0.0
133			200	0	0	0	0	0	0	94	0.0	0.0
200			200	0	0	0	0	0	0	53	0.0	0.0
PC: MMC			200	9	43	0	0	0	0	87	26.0*	0.0
NC	6-18	+	200	0	0	0	0	0	0	100	0.0	0.0
26.3			200	0	2	0	0	0	0	83	1.0	0.0
39.5			200	2	24	0	0	0	0	60	13.0*	0.0
59.3			200	22	119	0	0	0	0	32	64.0*	0.0
88.9			200	13	119	0	0	0	0	26	62.0*	0.0
PC: CP			200	10	109	0	0	0	0	67	57.5*	0.0

a) ctb: chromatid break, cte: chromatid exchange, csb: chromosome break, cse: chromosome exchange, other: including fragmentation, g: chromatid or chromosome gap, RCC: Relative Cell Count

NC: Negative control (DMSO), PC: Positive Control; CP: Cyclophosphamide (14.0), MMC: Mitomycin C (0.075)

*: The frequency was more than 10%.

Table S1-22 In vitro mammalian chromosomal aberration test of Butyl 2-naphthyl ether（Negative）

Butyl 2-naphthyl ether (μg/mL)	Time (h)	S9 mix	Number of cells with structural chromosome aberration[(a)]							RICC (%)	Structural aberrations (%)	Numerical aberrations (%)
			Observed cells	Chromatid type		Chromosome type		Others	g			
				ctb	cte	csb	cse					
NC	6-18	-	300	1	0	0	0	0	0	100	0.3	0.3
10			300	2	2	0	0	0	1	93	1.0	0.3
20			300	2	0	0	0	0	1	72	0.7	0.7
30			300	0	0	0	0	0	0	75	0.0	0.3
40			300	1	0	0	0	0	2	59	0.3	0.0
50			300	0	0	0	0	0	2	42	0.0	1.3
PC: MMC			300	24	93	4	2	0	2	62	37.0*	1.0
NC	6-18	+	300	0	0	0	0	0	0	100	0.0	1.0
2.5			300	0	1	0	0	0	1	109	0.3	0.7
5.0			300	0	0	0	0	0	0	83	0.0	0.0
10			300	0	0	0	0	0	0	47	0.0	0.3
20			300	5	12	3	1	0	2	21	5.7*	1.0
PC: B[a]P			300	20	162	1	8	0	1	31	56.7*	0.3
NC	24	-	300	1	0	0	0	0	0	100	0.3	0.3
2.5			300	0	0	0	0	0	0	109	0.0	0.3
5.0			300	0	1	0	0	0	0	82	0.3	0.7
10			300	1	1	0	0	0	2	68	0.3	0.3
20			300	1	0	0	0	0	0	58	0.3	0.7
40			300	2	1	0	0	0	1	45	1.0	1.0
PC: MMC			300	35	118	3	1	0	1	73	46.7*	0.3

a) ctb: chromatid break, cte: chromatid exchange, csb: chromosome break, cse: chromosome exchange, other: including fragmentation, g: chromatid or chromosome gap, RICC: Relative Increase Cell Count

NC: Negative control (DMSO), PC: Positive Control; B[a]P: Benzo[a]pyrene (20.0), MMC: Mitomycin C (0.1)

*: $p < 0.05$ (significant difference by Fisher’s exact test)

Table S1-23 In vitro mammalian chromosomal aberration test of Vanillin propyleneglycol acetal（Positive）

Vanillin propyleneglycol acetal (μg/mL)	Time (h)	S9 mix	Number of cells with structural chromosome aberration[a]							RICC (%)	Structural aberrations (%)	Numerical aberrations (%)
			Observed cells	Chromatid type		Chromosome type		Others	g			
				ctb	cte	csb	cse					
NC	6-18	-	200	1	2	0	0	0	0	100	1.5	0.0
1700			200	3	3	0	0	0	1	73.5	3.0	1.5
1900			200	9	5	0	0	0	0	67.2	6.0	0.0
2100			200	26	25	0	0	0	3	50.8	18.0*	0.0
PC: MMC			200	91	78	0	0	0	2	No data	63.5*	0.0
NC	6-18	+	200	0	0	0	0	0	0	100	0.0	0.5
131			200	3	3	0	0	0	1	78.5	2.5	1.0
263			200	17	18	1	0	0	0	54.5	14.0*	0.5
525			200	20	33	0	0	0	0	46.2	20.0*	1.0
PC: CP			200	35	55	0	0	0	1	No data	40.0*	0.0

a) ctb: chromatid break, cte: chromatid exchange, csb: chromosome break, cse: chromosome exchange, other: including fragmentation, g: chromatid or chromosome gap, RICC: Relative Increase Cell Count

NC: Negative control (DMSO), PC: Positive Control; CP: cyclophosphamide (6.0), MMC: Mitomycin C (0.1)

*: The frequency was more than 10%.

Table S1-24 In vitro mammalian chromosomal aberration test of 4-ethenyl-2-methoxyphenol（Positive）

4-ethenyl-2-methoxyphenol (μg/mL)	Time (h)	S9 mix	Number of cells with structural chromosome aberration[(a)] Observed cells	Chromatid type ctb	cte	Chromosome type csb	cse	Others	g	RICC (%)	Structural aberrations (%)	Numerical aberrations (%)
NC	6-18	-	300	2	0	0	0	0	1	100	0.7	1.0
19			300	3	2	0	0	0	3	91	1.7	0.3
38			300	3	4	1	0	0	1	82	2.7	0.3
75			300	6	13	0	0	0	0	76	5.0*	1.7
150			300	5	25	0	1	0	0	62	8.3*	1.0
300			300	15	52	1	0	0	0	49	17.3*	1.3
PC: MMC			300	30	77	4	3	0	1	63	31.3*	1.0
NC	6-18	+	300	1	0	0	1	0	0	100	0.7	0.0
38			300	0	0	0	0	0	1	80	0.0	0.3
75			300	0	1	0	0	0	0	76	0.3	0.7
150			300	0	5	0	0	0	0	55	1.7	1.0
300			300	1	25	0	0	0	0	45	8.3*	0.7
PC: B[a]P			300	25	161	4	6	0	2	47	56.3*	0.7
NC	24	-	300	2	1	0	1	0	1	100	1.0	0.0
3.0			300	0	0	0	0	0	0	90	0.0	0.7
5.0			300	2	3	0	0	0	2	77	1.3	0.0
10			300	5	3	1	0	0	0	44	2.3	0.0
20			300	7	5	2	0	0	2	49	4.7*	1.0
40			300	7	13	19	7	0	1	44	12.3*	0.3
PC: MMC			300	27	78	3	1	0	0	81	32.3*	0.3

a) ctb: chromatid break, cte: chromatid exchange, csb: chromosome break, cse: chromosome exchange, other: including fragmentation, g: chromatid or chromosome gap, RICC: Relative Increase Cell Count

NC: Negative control (DMSO), PC: Positive Control; B[a]P: Benzo[a]pyrene (20.0), MMC: Mitomycin C (0.1)

*: $p < 0.05$ (significant difference by Fisher's exact test)

Table S1-25 In vitro mammalian chromosomal aberration test of Furfural propyleneglycol acetal（Positive）

Furfural propyleneglycol acetal (μg/mL)	Time (h)	S9 mix	Observed cells	Chromatid type: ctb	Chromatid type: cte	Chromosome type: csb	Chromosome type: cse	Others	g	RICC (%)	Structural aberrations (%)	Numerical aberrations (%)
			Number of cells with structural chromosome aberration[(a)]									
NC	6-18	-	200	3	0	0	0	0	0	100	1.5	1.0
385			200	1	0	0	0	0	0	100.2	0.5	0.0
770			200	1	2	0	0	0	1	94.7	1.5	1.0
1540			200	2	1	0	0	0	0	86.9	1.5	1.0
PC: MMC			200	84	82	0	0	0	0	No data	65.5*	1.0
NC	6-18	+	200	0	0	0	0	0	1	100	0.0	0.5
12.0			200	1	0	0	0	0	0	91.9	0.5	0.0
17.0			200	8	3	0	0	0	2	64.8	4.5	0.5
24.1			200	37	29	0	0	0	0	24.0	23.5*	1.0
PC: CP			200	18	36	0	0	0	2	No data	25.5*	0.0
NC	6-18	+	200	1	1	0	0	0	1	100	1.0	0.0
14.1			200	5	1	0	0	0	0	79.4	3.0	0.0
16.8			200	13	11	0	0	0	4	54.5	10.0*	0.5
20.0			200	37	21	0	0	0	1	43.4	22.5*	1.5
PC: CPA			200	14	32	0	0	0	2	No data	22.5*	0.5

a) ctb: chromatid break, cte: chromatid exchange, csb: chromosome break, cse: chromosome exchange, other: including fragmentation, g: chromatid or chromosome gap, RICC: Relative Increase Cell Count

NC: Negative control (DMSO), PC: Positive Control; CP: Cyclophosphamide (6.0), MMC: Mitomycin C (0.1)

*: The frequency was more than 10%.

Table S1-26 In vitro mammalian chromosomal aberration test of 5-methyl-2-furfural（Positive）

5-methyl-2-furfural (μg/mL)	Time (h)	S9 mix	Number of cells with structural chromosome aberration[(a)] Observed cells	Chromatid type ctb	cte	Chromosome type csb	cse	Others	g	RPD (%)	Structural aberrations (%)	Numerical aberrations[(b)] (%)
NC	6-18	-	300	2	0	0	0	0	1	100	0.7	0.8
300			300	4	2	1	0	0	2	95.6	2.0	0.5
600			300	5	0	0	0	0	0	80.4	1.7	1.5
1200			300	2	2	1	0	0	3	70.0	1.7	8.0*
PC: MMC			300	21	14	2	0	0	0	No data	12.3*	0.0
NC	6-18	+	300	2	0	0	0	0	0	100	0.7	0.8
104			300	3	3	0	0	0	0	108.1	2.0	3.3*
125			300	3	0	1	0	0	0	63.0	1.3	3.5*
150			300	3	6	2	0	0	1	42.4	3.3*	7.8*
PC: CP			300	17	44	2	0	0	1	No data	19.3*	0.3
NC	24-0	-	300	1	0	0	0	0	0	100	0.3	0.0
417			300	2	0	1	0	0	0	83.9	1.0	1.0
500			300	2	0	1	0	0	0	50.8	1.0	0.0
600			300	4	3	1	0	0	1	53.1	2.7*	1.3*
720			300	2	1	0	0	0	0	47.5	1.0	1.0
PC: MMC			300	18	49	1	0	0	0	No data	20.0*	0.0

a) ctb: chromatid break, cte: chromatid exchange, csb: chromosome break, cse: chromosome exchange, other: including fragmentation, g: chromatid or chromosome gap, RPD: Relative Population Doubling

b) Observed cells were 400.

NC: Negative control (DMSO), PC: Positive Control; CP: cyclophosphamide (5.0), MMC: Mitomycin C (0.05)

*: $p < 0.05$ (significant difference by $\chi 2$ test)

Table S1-27 In vitro mammalian chromosomal aberration test of 2-(4-methyl-5-thiazolyl)ethanol（Negative）

2-(4-methyl-5-thiazolyl)ethanol (μg/mL)	Time (h)	S9 mix	Number of cells with structural chromosome aberration[(a)] Observed cells	Chromatid type ctb	cte	Chromosome type csb	cse	Others	g	RCC (%)	Structural aberrations (%)	Numerical aberrations (%)
NC	6-18	-	200	1	0	0	0	0	1	100	0.5	0.5
358			200	2	0	0	0	0	0	87.2	1.0	0.0
715			200	2	1	0	0	0	1	91.9	1.5	0.5
1430			200	0	2	0	0	0	1	78.9	1.0	0.0
PC: MMC(1)			200	90	78	0	0	0	1	No data	62.5*	1.5
NC	6-18	+	200	1	0	0	0	0	0	100	0.5	0.5
358			200	1	0	0	0	0	0	96.9	0.5	0.0
715			200	0	2	0	0	0	0	89.8	1.0	0.0
1430			200	0	0	0	0	0	1	81.4	0.0	0.0
PC: CP			200	30	41	0	1	0	0	No data	33.5*	0.0
NC	24	-	200	1	1	0	0	0	1	100	1.0	0.0
358			200	2	0	0	0	0	0	90.7	1.0	0.5
715			200	1	0	0	0	0	0	76.9	0.5	0.5
1430			200	1	2	0	0	0	0	63.3	1.5	0.0
PC: MMC(2)			200	85	94	0	0	0	3	No data	67.5*	0.0

a) ctb: chromatid break, cte: chromatid exchange, csb: chromosome break, cse: chromosome exchange, other: including fragmentation, g: chromatid or chromosome gap, RCC: Relative Cell Count

NC: Negative control (distilled water), PC: Positive Control; CP: Cyclophosphamide (6.0), MMC: Mitomycin C ((1)0.1,(2)0.05)

*: The frequency was more than 10%.

Table S1-28 In vitro mammalian chromosomal aberration test of 5-methyl-2-phenyl-2-hexenal（Positive）

5-methyl-2-phenyl-2-hexenal (μg/mL)	Time (h)	S9 mix	Number of cells with structural chromosome aberration[(a)]							RCC	Structural aberrations (%)	Numerical aberrations (%)
			Observed	Chromatid type		Chromosome type						
			cells	ctb	cte	csb	cse	Others	g	(%)		
NC	6-18	-	200	2	2	0	0	0	0	100	2.0	0.0
60.0			200	0	0	0	0	0	1	68.2	0.0	0.5
70.0			200	5	0	0	0	0	0	58.1	2.5	1.5
80.0			200	17	0	0	0	0	0	49.5	8.5	0.5
PC: MMC[(1)]			200	81	75	0	0	0	1	No data	58.5*	1.5
NC	6-18	+	200	1	1	0	0	0	0	100	1.0	1.0
140			200	2	3	0	0	0	1	79.7	2.5	1.5
160			200	2	1	0	0	0	0	74.3	1.0	1.0
180			200	2	3	0	0	0	2	53.6	2.5	0.5
PC: CP			200	26	23	0	1	0	1	No data	24.5*	0.5
NC	24	-	200	5	0	0	0	0	0	100	2.5	0.5
40.0			200	10	1	0	0	0	2	69.7	5.5	3.0
50.0			200	16	0	0	1	0	1	52.1	8.0	0.0
60.0			200	23	0	0	0	0	0	45.7	11.5*	1.0
PC: MMC[(2)]			200	78	75	0	0	0	4	No data	61.0*	1.0

a) ctb: chromatid break, cte: chromatid exchange, csb: chromosome break, cse: chromosome exchange, other: including fragmentation, g: chromatid or chromosome gap, RCC: Relative Cell Count

NC: Negative control (DMSO), PC: Positive Control; CP: Cyclophosphamide (6.0), MMC: Mitomycin C ((1)0.1,(2) 0.05)

*: The frequency was more than 10%.

Table S1-29 In vitro mammalian chromosomal aberration test of 4-methylbenzaldehyde（Positive）

4-methylbenzaldehyde (μg/mL)	Time (h)	S9 mix	Number of cells with structural chromosome aberration[a]							RICC (%)	Structural aberrations (%)	Numerical aberrations (%)
			Observed cells	Chromatid type		Chromosome type		Others	g			
				ctb	cte	csb	cse					
NC	6-18	-	300	1	2	0	0	0	1	100	1.0	0.3
75			300	3	3	0	0	0	1	89	1.7	0.7
150			300	4	1	0	0	0	1	61	2.0	1.7
300			300	4	2	0	0	0	2	59	2.3	1.7
600			300	6	10	0	0	0	2	38	4.3*	0.0
PC: MMC			300	31	104	0	0	0	0	38	37.7*	0.3
NC	6-18	+	300	0	2	0	0	0	0	100	0.7	0.0
75			300	1	1	0	0	0	0	100	0.7	0.3
150			300	1	0	0	1	0	0	85	0.7	0.3
300			300	0	1	0	0	0	0	63	0.3	0.0
600			300	0	0	0	0	0	1	47	0.0	0.7
PC: B[a]P			300	20	118	0	0	0	1	64	41.0*	1.0
NC	24	-	300	3	0	0	0	0	1	100	1.0	0.3
25			300	2	0	1	0	0	2	96	1.7	0.0
50			300	2	0	1	0	0	1	96	1.0	0.7
100			300	4	1	0	0	0	5	68	2.7	0.7
200			300	7	2	2	0	0	5	46	3.7*	0.3
PC: MMC			300	58	124	3	3	0	2	64	49.0*	0.3

a) ctb: chromatid break, cte: chromatid exchange, csb: chromosome break, cse: chromosome exchange, other: including fragmentation, g: chromatid or chromosome gap, RICC: Relative Increase Cell Count

NC: Negative control (DMSO), PC: Positive Control; B[a]P: Benzo[a]pyrene (20.0), MMC: Mitomycin C (0.1)

*: $p < 0.05$ (significant difference by Fisher's exact test)

Table S1-30 In vitro mammalian chromosomal aberration test of Delta-dodecalactone（Negative）

Delta-dodecalactone (μg/mL)	Time (h)	S9 mix	Observed cells	Chromatid type: ctb	Chromatid type: cte	Chromosome type: csb	Chromosome type: cse	Others	g	RCC (%)	Structural aberrations (%)	Numerical aberrations (%)
			Number of cells with structural chromosome aberration[(a)]									
NC	6-18	-	200	3	1	0	0	0	2	100	2.0	0.0
125			200	3	0	0	0	0	2	93	1.5	0.5
250			200	2	0	0	0	0	2	85	1.0	0.0
500			200	1	0	0	0	0	3	66	0.5	0.5
1000			200	0	0	0	0	0	3	57	0.0	0.0
2000			200	1	2	0	0	0	0	50	1.5	0.0
PC: MMC[(1)]			200	41	40	0	0	0	1	82	33.0*	0.0
NC	6-18	+	200	1	0	0	0	0	1	100	0.5	0.0
125			200	1	1	0	0	0	1	100	1.0	0.0
250			200	3	1	0	0	0	2	71	2.0	0.5
500			200	1	0	0	0	0	1	64	0.5	0.5
1000			200	0	0	0	0	0	1	49	0.0	0.5
PC: CP			200	28	44	1	0	0	0	81	33.5*	0.0
NC	24	-	200	2	0	0	0	0	2	100	1.0	0.0
62.5			200	1	0	0	1	0	2	85	1.0	0.0
125			200	1	1	0	0	0	2	83	1.0	0.0
250			200	2	1	0	0	0	2	73	1.5	0.0
500			200	3	0	0	0	0	3	49	1.5	0.5
PC: MMC[(2)]			200	47	65	0	0	0	3	76	46.0*	0.0

a) ctb: chromatid break, cte: chromatid exchange, csb: chromosome break, cse: chromosome exchange, other: including fragmentation, g: chromatid or chromosome gap, RCC: Relative Cell Count

NC: Negative control (DMSO), PC: Positive Control; CP: Cyclophosphamide (14.0), MMC: Mitomycin C ((1)0.075,(2)0.05)

*: The frequency was more than 10%.
