# Supplementary material for: Genotoxicity assessment of food-flavoring chemicals used in Japan
Source: Toxicol Rep. 2022 Apr 27;9:1008–12. doi: 10.1016/j.toxrep.2022.04.026 (PMC9742862; doi:10.1016/j.toxrep.2022.04.026)
Supplement: Supplementary file 2 — Supplementary material [file mmc2.pdf]

Table S2-1 (1) The results of micronucleus test in bone marrow of male CD1 mice after 5-hexenyl isothiocyanate treatment （Negative）

Compound	Dose (mg/kg/day)	No. of Animal	% MNPCE	% PCE
Control	0	5	0.16 ±0.09	54.7 ±2.6
5-hexenyl	62.5	5	0.11 ±0.05	47.4 ±6.4
isothiocyanate	125	5	0.16 ±0.05	45.6 ±7.0*
	250	5	0.11 ±0.05	43.6 ±4.5*
MMC	1	5	2.84 ±0.73	45.0 ±8.3

No significant difference between the negative control group and any treated group (Fisher's exact test and Cochran-Armitage test)
*:p<0.05, significant difference from control (Dunnett’s test)
Control: corn oil (10 ml/kg)
Positive control: MMC (1 mg/kg, single i.p.)

Table S2-1 (11). The results of micronucleus test in bone marrow of male CD1 mice after 2-methyl butyric acid treatment (Negative)

Compound	Dose (mg/kg/day)	No. of Animal	% MNPCE	% PCE
Control	0	5	0.128 ±0.053	50.10 ±2.14
2-Methyl	500	5	0.116 ±0.055	49.70 ±1.91
butyric acid	1000	5	0.112 ±0.039	49.42 ±1.98
	2000	5	0.138 ±0.029	51.48 ±2.53
MMC	2	5	2.806 ±1.014 *	46.74 ±3.21

Based on 4,000 polychromatic erythrocytes per animal.
No significant difference between the negative control group and any treated group (Kastenbaum and Bowman test and Cochran-Armitage test)
*:p<0.05, significant difference from control (Kastenbaum and Bowman)
Control: olive oil (10 ml/kg)
Positive control: MMC (2 mg/kg, single i.p.)

Table S2-3 (13) The results of micronucleus test in bone marrow of male CD1 mice after 2-hexenol treatment （Negative）

**Compound**	**Dose (mg/kg/day)**	**No. of Animal**	**% MNPCE**	**% PCE**
Control	0	5	0.15 ±0.04	56.4 ±7.2
trans-2-hexenol	250	5	0.15 ±0.04	55.2 ±9.9
	500	5	0.09 ±0.01	50.6 ±2.1
	1000	5	0.10 ±0.08	55.6 ±6.3
MMC	1	5	2.47 ±0.59*	54.6 ±6.7

No significant difference between the negative control group and any treated group (Fisher's exact test and Cochran-Armitage test)
*:p<0.05, significant difference from control (Fisher's exact test)
Control: corn oil (10 ml/kg)
Positive control: MMC (1 mg/kg, single i.p.)

Table S2-4 (17) The results of micronucleus test in bone marrow of male CD1 mice after 1,3,5-undecatriene treatment（Negative）

**Compound**	**Dose (mg/kg/day)**	**No. of Animal**	**% MNPCE**	**% PCE**
Control	0	5	0.25 ±0.14	61.0 ±2.4
1,3,5-Undecatriene	125	5	0.19 ±0.07	61.3 ±5.5
	250	5	0.20 ±0.05	60.4 ±4.3
	500	5	0.21 ±0.11	60.1 ±7.3
MMC	2	5	7.92 ±0.76**	56.1 ±3.3[#]

**:$p<0.01$, significant difference from control (Fisher's exact test)
[#]:$p<0.05$, significant difference from control (Student’s t test)
Control: corn oil (10 ml/kg)
Positive control: MMC (2 mg/kg, single i.p.)

Table S2-5 (19) The results of micronucleus test in bone marrow of male CD1 mice after 2-furanmethanethiol treatment（Negative）

Compound	Dose (mg/kg/day)	No. of Animal	% MNPCE	% PCE
Control	0	5	0.17 ±0.08	54.0 ±2.7
2-furanmethanethiol	62.5	5	0.15 ±0.04	51.0 ±6.6
	125	5	0.09 ±0.04	51.6 ±5.6
	250	5	0.16 ±0.11	44.0 ±5.1*
MMC	1	5	2.60 ±0.81	49.1 ±2.8

No significant difference between the negative control group and any treated group (Fisher's exact test and Cochran-Armitage test)
*:p<0.05, significant difference from control (Dunnett’s test)
Control: corn oil (10 ml/kg)
Positive control: MMC (1 mg/kg, single i.p.)

Table S2-6 (21) The results of micronucleus test in bone marrow of CD1 mice after isoeugenyl methyl ether treatment（Negative）

Compound	Dose (mg/kg)	No. of Animal	% MNPCE	% PCE
Control	0	5	0.15 ± 0.03	55.1 ± 6.1
isoeugenyl methyl	125	5	0.12 ± 0.05	55.2 ± 7.7
ether	250	5	0.14 ± 0.08	57.3 ± 6.4
	500	5	0.17 ± 0.04	46.0 ± 4.7
MMC	1	5	3.26 ± 1.06	53.6 ± 7.3

No statistically significant difference in any test article administration group from negative control group.
Control: negative control (Corn oil, 10 ml/kg)
MMC: Positive control (Mitomycin C, dose only once , *i.p.*, 1 days after administration)

Table S2-7 (23) The results of micronucleus test in bone marrow of CD1 mice after vanillin propyleneglycol acetal (PGA) treatment（Negative）

**Compound**	**Dose (mg/kg)**	**No. of Animal**	**% MNPCE**	**% PCE**
Control	0	5	0.15 ± 0.11	57.4 ± 7.6
vanillin PGA	250	5	0.14 ± 0.15	68.1 ± 4.5[#]
	500	5	0.20 ± 0.18	68.1 ± 5.7[#]
	1000	5	0.15 ± 0.17	72.0 ± 3.7[##]
MMC	2	5	7.68 ± 1.24**	59.9 ± 1.7

**: $p<0.01$, significant difference from negative control (Fisher's exact test)
[#]: $p<0.05$, [##]: $p<0.01$, significantly different from between the negative control and each test article-treated group (Dunnett's test)
Control: negative control (Corn oil, 10 ml/kg)
MMC: Positive control (Mitomycin C, dose only once , *i.p.*, 1 days after administration)

Table S2-8 (24) The results of micronucleus test in bone marrow of male CD1 mice after 4-ethenyl-2-methoxyphenol treatment (Negative)

**Compound**	**Dose (mg/kg/day)**	**No. of Animal**	**% MNPCE**	**% PCE**
Control	0	5	0.122 ±0.041	49.64 ±2.24
4-ethenyl-2-	125	5	0.132 ±0.030	49.10 ±1.76
methoxyphenol	250	5	0.116 ±0.050	49.42 ±2.87
	500	5	0.162 ±0.036	50.46 ±1.71
MMC	2	5	2.586 ±0.459 *	46.88 ±1.86

Based on 4,000 polychromatic erythrocytes per animal.
No significant difference between the negative control group and any treated group (Kastenbaum and Bowman test and Cochran-Armitage test)
*:p<0.05, significant difference from control (Kastenbaum and Bowman)
Control: corn oil (10 ml/kg)
Positive control: MMC (2 mg/kg, single i.p.)

Table S2-9 (26) The results of micronucleus test in bone marrow of male F344 rats after 5-methyl-2-furfural treatment（Negative）

Compound	Dose (mg/kg/day)	No. of Animal	% MNPCE	% PCE
Control	0	5	0.16 ±0.05	28.8 ±7.1
5-methyl-2-furfural	250	5	0.17 ±0.10	27.9 ±5.0
	500	5	0.15 ±0.05	28.8 ±4.9
	1000	5	0.16 ±0.05	28.4 ±4.3
MMC	1	5	1.51 ±0.27**	31.4 ±8.5

**:p<0.01, significant difference from control (Kastenbaum and Bowman method)
Control: olive oil (10 ml/kg)
Positive control: MMC (1 mg/kg, single i.p.)

Table S2-10 (28) The results of micronucleus test in bone marrow of male CD1 mice after 5-methyl-2-phenyl-2-hexenal treatment（Negative）

Compound	Dose (mg/kg/day)	No. of Animal	% MNPCE	% PCE
Control	0	5	0.15 ±0.09	55.2 ±8.3
5-Methyl-2-phenyl-	250	5	0.13 ±0.02	50.7 ±8.4
2-hexenal	500	5	0.20 ±0.07	53.8 ±7.1
	1000	5	0.18 ±0.07	51.1 ±5.6
MMC	1	5	3.92 ±0.74*	55.2 ±5.3

No significant difference between the negative control group and any treated group (Fisher's exact test and Cochran-Armitage test)
*:p<0.05, significant difference from control (Fisher’s exact test)
Control: corn oil (10 ml/kg)
Positive control: MMC (1 mg/kg, single i.p.)

Table S2-11 (29) The results of micronucleus test in bone marrow of male CD1 mice after 4-methylbenzaldehyde treatment (Negative)

Compound	Dose (mg/kg/day)	No. of Animal	% MNPCE	% PCE
Control	0	5	0.122 ± 0.052	50.46 ± 1.15
4-Methyl	250	5	0.150 ± 0.071	49.90 ± 0.65
benzaldehyde	500	5	0.142 ± 0.029	50.08 ± 1.99
	1000	5	0.122 ± 0.041	49.96 ± 0.40
MMC	2	5	2.988 ± 0.581 *	43.00 ± 4.08 #

Based on 4,000 polychromatic erythrocytes per animal.
No significant difference between the negative control group and any treated group (Kastenbaum and Bowman test and Cochran-Armitage test)
*:p<0.05, significant difference from control (Kastenbaum and Bowman)
#:p<0.05, significant difference from control (Welch’s t-test)
Control: olive oil (10 ml/kg)
Positive control: MMC (2 mg/kg, single i.p.)
