# Supplementary material for: Genotoxicity assessment of food-flavoring chemicals used in Japan
Source: Toxicol Rep. 2022 Apr 27;9:1008–12. doi: 10.1016/j.toxrep.2022.04.026 (PMC9742862; doi:10.1016/j.toxrep.2022.04.026)
Supplement: Supplementary file 3 — Supplementary material [file mmc3.pdf]

Table S3-1A (9) The results of TGR assay in liver of Muta™ Mouse after 2,3-pentanedione treatment (Negative)

Compound	Dose (mg/kg)	Animal ID No.	Number of plaque forming units	Number of mutant Plaques	Mutant Frequency ($X10^{-6}$)	Mutant Frequency ($X10^{-6}$) Average ± S.D.
Control	0	1001	521,100	15	28.8	
		1002	616,500	22	35.7	
		1003	663,300	56	84.4	
		1004	549,000	27	49.2	
		1005	393,300	10	25.4	44.7± 24.0
2,3-pentanedione	125	1201	493,200	16	32.4	
		1202	733,500	70	95.4	
		1203	887,400	21	23.7	
		1204	547,200	24	43.9	
		1205	440,100	12	27.3	44.5 ± 29.4
	250	1301	390,600	13	33.3	
		1302	441,900	21	47.5	
		1303	510,300	18	35.3	
		1304	401,400	11	27.4	
		1305	396,900	21	52.9	39.3± 10.6
	500	1401	341,100	10	29.3	
		1403	763,200	29	38.0	
		1404	470,700	20	42.5	
		1405	412,200	18	43.7	
		1406	457,200	17	37.2	38.1 ± 5.7
ENU	100	1501	895,500	126	140.7	
		1502	436,500	40	91.6	
		1503	311,400	44	141.3	
		1504	368,100	38	103.2	
		1505	428,400	49	114.4	118.2 ± 22.3 *

*:$p<0.05$, significant difference from negative control (Student's t test)
Control: negative control (Corn oil, 10 ml/kg)
ENU: Positive control (*N*-ethyl-*N*-nitrosourea, 10 ml/kg, *i.p.*, dose once a day, for 2 days, expression period; 10 days)

Table S3-1B (9) The results of TGR assay in glandular stomach of Muta^{TM} Mouse after 2,3-pentanedione treatment (Negative)

Compound	Dose (mg/kg)	Animal ID No.	Number of plaque forming units	Number of mutant Plaques	Mutant Frequency (X10^{-6})	Mutant Frequency (X10^{-6}) Average ± S.D.
Control	0	1001	520,200	19	36.5	
		1002	626,400	30	47.9	
		1003	555,300	16	28.8	
		1004	703,800	22	31.3	
		1005	342,900	14	40.8	37.1± 7.6
2,3-pentanedione	125	1201	550,800	22	39.9	
		1202	563,400	24	42.6	
		1203	716,400	16	22.3	
		1204	444,600	18	40.5	
		1205	413,100	15	36.3	36.3 ± 8.2
	250	1301	452,700	23	50.8	
		1302	656,100	27	41.2	
		1303	468,000	13	27.8	
		1304	674,100	27	40.1	
		1305	657,900	17	25.8	37.1 ± 10.3
	500	1401	409,500	25	61.1	
		1403	475,200	10	21.0	
		1404	671,400	28	41.7	
		1405	943,200	38	40.3	
		1406	675,900	24	35.5	39.9 ± 14.4
ENU	100	1501	514,800	216	419.6	
		1502	536,400	223	415.7	
		1503	528,300	198	374.8	
		1504	520,200	230	442.1	
		1505	385,200	156	405.0	411.4 ± 24.5 *

*:$p<0.05$, significant difference from negative control (Aspin-Welch's t test)
Control: negative control (Corn oil, 10 ml/kg)
ENU: Positive control (*N*-ethyl-*N*-nitrosourea, 10 ml/kg, *i.p.*, dose once a day, for 2 days, expression period; 10 days)

Table S3-2B (10) The results of TGR assay in liver of Muta™ Mouse after raspberry ketone treatment（Negative）

Compound	Dose (mg/kg)	Animal ID No.	Number of plaque forming units	Number of mutant Plaques	Mutant Frequency ($X10^{-6}$)	Mutant Frequency ($X10^{-6}$) Average ± S.D.
Control	0	3001	1,152,900	84	72.9	
		3002	783,900	29	37.0	
		3003	970,200	35	36.1	
		3004	858,600	35	40.8	
		3005	997,200	51	51.1	47.6 ± 15.4
raspberry ketone	250	3201	1,458,900	52	35.6	
		3202	1,161,000	58	50.0	
		3203	1,660,500	85	51.2	
		3204	1,038,600	62	59.7	
		3205	1,152,000	51	44.3	48.2 ± 8.9
	500	3301	863,100	70	81.1	
		3302	783,000	45	57.5	
		3303	503,100	37	73.5	
		3304	1,413,000	104	73.6	
		3305	1,006,200	49	48.7	66.9 ± 13.3
	1000	3401	1,195,200	55	46.0	
		3402	707,400	34	48.1	
		3403	746,100	43	57.6	
		3404	419,400	29	69.1	
		3405	1,100,700	24	21.8	48.5 ± 17.5
ENU	100	3501	634,500	102	160.8	
		3502	709,200	145	204.5	
		3503	530,100	78	147.1	
		3504	365,400	78	213.5	
		3505	930,600	223	239.6	193.1 ± 38.3*

*:p<0.05, significant difference from negative control (Student's t test)
Control: negative control (Corn oil, 10 ml/kg)
ENU: Positive control (*N*-ethyl-*N*-nitrosourea, 10 ml/kg, *i.p.*, dose once a day, for 2 days, expression period; 10 days)

Table S3-2B (10) The results of TGR assay in glandular stomach of Muta^{TM} Mouse after raspberry ketone treatment (Negative)

Compound	Dose (mg/kg)	Animal ID No.	Number of plaque forming units	Number of mutant Plaques	Mutant Frequency (X10^{-6})	Mutant Frequency (X10^{-6}) Average ± S.D.
Control	0	3001	730,800	40	54.7	
		3002	439,200	22	50.1	
		3003	382,500	19	49.7	
		3004	430,200	18	41.8	
		3005	557,100	23	41.3	47.5 ± 5.8
raspberry ketone	250	3201	589,500	26	44.1	
		3202	409,500	22	53.7	
		3203	718,200	43	59.9	
		3204	508,500	25	49.2	
		3205	470,700	19	40.4	49.5 ± 7.7
	500	3301	533,700	19	35.6	
		3302	500,400	13	26.0	
		3303	420,300	8	19.0	
		3304	580,500	32	55.1	
		3305	864,900	37	42.8	35.7 ± 14.1
	1000	3401	924,300	44	47.6	
		3402	598,500	24	40.1	
		3403	665,100	37	55.6	
		3404	1,148,400	75	65.3	
		3405	944,100	59	62.5	54.2 ± 10.4
ENU	100	3501	545,400	341	625.2	
		3502	688,500	467	678.3	
		3503	391,500	155	395.9	
		3504	657,000	340	517.5	
		3505	665,100	362	544.3	552.2 ± 108.3*

*:$p<0.05$, significant difference from negative control (Aspin-Welch's t test)
Control: negative control (Corn oil, 10 ml/kg)
ENU: Positive control (*N*-ethyl-*N*-nitrosourea, 10 ml/kg, *i.p.*, dose once a day, for 2 days, expression period; 10 days)

Table S3-3A (25) The results of TGR assay in liver of Muta™ Mouse after furfural propyleneglycol acetal treatment (Negative)

Compound	Dose (mg/kg)	Animal ID No.	Number of plaque forming units	Number of mutant Plaques	Mutant Frequency ($X10^{-6}$)	Mutant Frequency ($X10^{-6}$) Average ± S.D.
Control	0	3001	476,100	23	48.3	
		3002	552,600	26	47.1	
		3003	539,100	18	33.4	
		3004	434,700	22	50.6	
		3005	713,700	39	54.6	46.8 ± 8.0
furfural propyleneglycol acetal	25	3101	781,200	33	42.2	
		3102	426,600	20	46.9	
		3103	410,400	19	46.3	
		3104	371,700	19	51.1	
		3105	355,500	11	30.9	43.5 ± 7.7
	50	3201	530,100	25	47.2	
		3202	694,800	25	36.0	
		3203	590,400	22	37.3	
		3204	749,700	32	42.7	
		3205	803,700	41	51.0	42.8 ± 6.4
	100	3301	864,900	75	86.7	
		3302	383,400	35	91.3	
		3303	340,200	21	61.7	
		3304	538,200	21	39.0	
		3305	303,300	13	42.9	64.3 ± 24.2
ENU	100	3401	366,300	49	133.8	
		3402	668,700	78	116.6	
		3403	327,600	30	91.6	
		3404	695,700	110	158.1	
		3405	597,600	72	120.5	124.1 ± 24.4*

*:$p<0.05$, significant difference from negative control (Student's t test)
Control: negative control (Corn oil, 10 ml/kg)
ENU: Positive control (*N*-ethyl-*N*-nitrosourea, 10 ml/kg, *i.p.*, dose once a day, for 2 days, expression period; 10 days)

Table S3-3B (25) The results of TGR assay in glandular stomach of Muta™ Mouse after furfural propyleneglycol acetal treatment (Negative)

Compound	Dose (mg/kg)	Animal ID No.	Number of plaque forming units	Number of mutant Plaques	Mutant Frequency ($X10^{-6}$)	Mutant Frequency ($X10^{-6}$) Average ± S.D.
Control	0	3001	514,800	43	83.5	
		3002	367,200	18	49.0	
		3003	504,900	21	41.6	
		3004	580,500	25	43.1	
		3005	372,600	18	48.3	53.1 ± 17.3
furfural propyleneglycol acetal	25	3101	545,400	21	38.5	
		3102	709,200	34	47.9	
		3103	378,000	15	39.7	
		3104	643,500	29	45.1	
		3105	437,400	24	54.9	45.2 ± 6.6
	50	3201	559,800	24	42.9	
		3202	740,700	38	51.3	
		3203	387,000	22	56.8	
		3204	507,600	22	43.3	
		3205	576,900	27	46.8	48.2 ± 5.9
	100	3301	392,400	16	40.8	
		3302	475,200	23	48.4	
		3303	529,200	31	58.6	
		3304	451,800	22	48.7	
		3305	606,600	29	47.8	48.9 ± 6.3
ENU	100	3401	387,900	142	366.1	
		3402	791,100	349	441.2	
		3403	586,800	210	357.9	
		3404	416,700	143	343.2	
		3405	420,300	156	371.2	375.9 ± 38.0*

*:$p<0.05$, significant difference from negative control (Student's t test)
Control: negative control (Corn oil, 10 ml/kg)
ENU: Positive control (*N*-ethyl-*N*-nitrosourea, 10 ml/kg, *i.p.*, dose once a day, for 2 days, expression period; 10 days)
